# Development of a clinical prediction model for benign and malignant pulmonary nodules with a CTR ≥ 50% utilizing artificial intelligence-driven radiomics analysis

**DOI:** 10.1186/s12880-024-01533-9

**Published:** 2025-01-17

**Authors:** Wensong Shi, Yuzhui Hu, Guotao Chang, He Qian, Yulun Yang, Yinsen Song, Zhengpan Wei, Liang Gao, Hang Yi, Sikai Wu, Kun Wang, Huandong Huo, Shuaibo Wang, Yousheng Mao, Siyuan Ai, Liang Zhao, Xiangnan Li, Huiyu Zheng

**Affiliations:** 1 Department of Thoracic Surgery, The Fifth Clinical Medical College of Henan, University of Chinese Medicine (Zhengzhou People’s Hospital), Zhengzhou, 450003 China; 2 Department of Thoracic Surgery, The First Affiliated Hospital of Zhengzhou University, Zhengzhou, 450052 China; 3 Department of Geratology, Ninth People’s Hospital of Zhengzhou, Zhengzhou, 450053 China; 4 Translational Medicine Research Center (Key Laboratory of Organ Transplantation of Henan Province), The Fifth Clinical Medical College of Henan University of Chinese Medicine (Zhengzhou People’s Hospital), Zhengzhou, China; 5 Department of Radiology, Ninth People’s Hospital of Zhengzhou, Zhengzhou, 450053 China; 6 Department of Thoracic Surgery, National Cancer Center/National Clinical Research Center for Cancer/Cancer Hospital, Chinese Academy of Medical Sciences and Peking Union Medical College, Beijing, 100021 China; 7 Department of Thoracic Surgery, Liangxiang Hospital, Beijing, 102401 Fangshan District China; 8 Shukun (Beijing) Technology Co, Beijing, 100102 China; 9 Department of Thoracic Surgery, Beijing Institute of Respiratory Medicine and Beijing Chao-Yang Hospital, Capital Medical University, Beijing, 100006 China

**Keywords:** Artificial intelligence, Radiomics, 3D CTR ≥ 50%, Benign and malignant, Clinical prediction model

## Abstract

**Objective:**

In clinical practice, diagnosing the benignity and malignancy of solid-component-predominant pulmonary nodules is challenging, especially when 3D consolidation-to-tumor ratio (CTR) ≥ 50%, as malignant ones are more invasive. This study aims to develop and validate an AI-driven radiomics prediction model for such nodules to enhance diagnostic accuracy.

**Methods:**

Data of 2,591 pulmonary nodules from five medical centers (Zhengzhou People’s Hospital, etc.) were collected. Applying exclusion criteria, 370 nodules (78 benign, 292 malignant) with 3D CTR ≥ 50% were selected and randomly split 7:3 into training and validation cohorts. Using R programming, Lasso regression with 10-fold cross-validation filtered features, followed by univariate and multivariate logistic regression to construct the model. Its efficacy was evaluated by ROC, DCA curves and calibration plots.

**Results:**

Lasso regression picked 18 non-zero coefficients from 108 features. Three significant factors—patient age, solid component volume and mean CT value—were identified. The logistic regression equation was formulated. In the training set, the ROC AUC was 0.721 (95%CI: 0.642–0.801); in the validation set, AUC was 0.757 (95%CI: 0.632–0.881), showing the model’s stability and predictive ability.

**Conclusion:**

The model has moderate accuracy in differentiating benign from malignant 3D CTR ≥ 50% nodules, holding clinical potential. Future efforts could explore more to improve its precision and value.

**Clinical trial number:**

Not applicable.

## Introduction

**Fig. 1:**
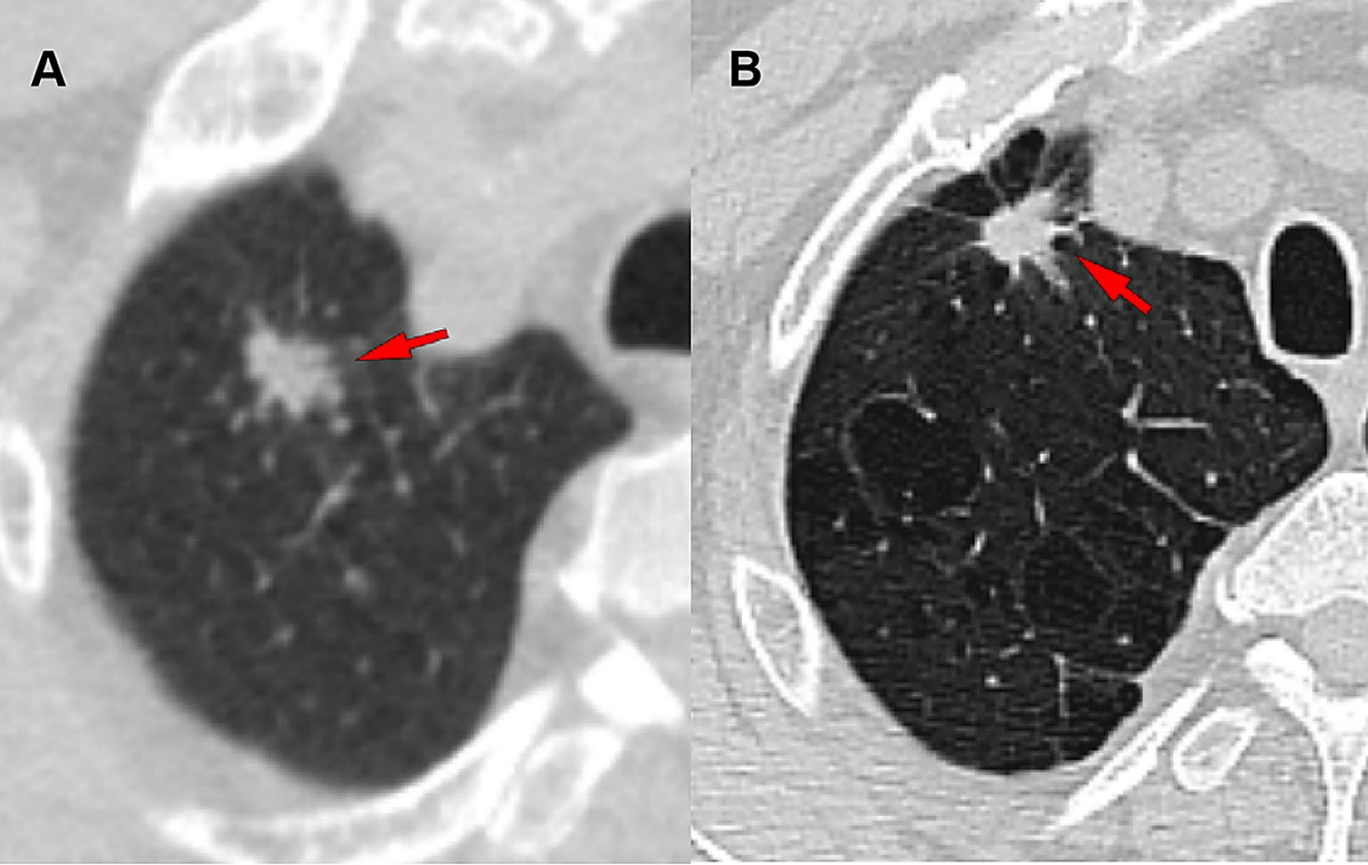
Two lung nodules (CTR > 50%) were detected in the right upper lobe. Surgical pathology verified one as benign and the other as malignant. (**A**) A 74-year-old male patient presented with a pulmonary nodule in the upper lobe of the right lung (indicated by the arrow in the imaging), which had a consolidation to tumor ratio of 85.5%. The nodule was surgically resected, and the postoperative pathological diagnosis revealed pulmonary tuberculosis. (**B**) A 59-year-old male patient presented with a pulmonary nodule in the upper lobe of the right lung (indicated by the arrow in the imaging), which had a consolidation to tumor ratio of 96.6%. The nodule was surgically resected, and the postoperative pathological diagnosis revealed pulmonary invasive adenocarcinoma

Pulmonary nodules are defined as focal, round opacities measuring less than or equal to 3 cm in diameter on chest imaging, with either clear or blurry edges, and can be a radiographic manifestation of a variety of diseases. Ground-glass nodules (GGNs) are an important type, which can be further classified into pure ground-glass nodules and mixed-density ground-glass nodules. Once a mixed-density ground-glass nodule is confirmed as malignant, its aggressiveness is often higher than that of pure ground-glass nodules. The more substantial the solid component, the faster the progression and the earlier the potential for metastasis, leading to a poorer prognosis [1, 2]. Differentiating the benignancy or malignancy of mixed-density ground-glass nodules with a predominant solid component (CTR ≥ 50%) is a clinical challenge(Fig. 1). Therefore, clarifying the nature of such nodules at the pulmonary nodule stage facilitates early treatment and is crucial for improving the prognosis of these patients. In clinical practice, a combination of methods is often employed to assess the nature of pulmonary nodules. These include patient history such as smoking habits and family history of cancer, radiographic morphological characteristics, and blood-based biomarkers. In some cases, a strategy of empirical anti-infective treatment followed by a reassessment of the changes in the nodule is also adopted to evaluate the malignancy. However, malignant nodules at the nodule stage often lack specific clinical manifestations, and morphological features such as spiculated margins and pleural indentation are not typical. Additionally, there is considerable overlap in the morphological characteristics of benign and malignant nodules, making differential diagnosis challenging.

Traditional tumor markers, such as Carcinoembryonic Antigen (CEA), Squamous Cell Carcinoma Antigen (SCC), Cytokeratin-19 Fragment Antigen 21 − 1 (CYFRA 21 − 1), and Gastrin-releasing Peptide Precursor (ProGRP), have limited sensitivity and specificity in diagnosing small nodules due to their limited secretion capabilities [3–5]. Hence, there is an urgent need for a novel diagnostic method to assist clinical decision-making. The traditional diagnostic model of pulmonary nodules relies on morphological features of pulmonary nodules. But these mainly depends on the subjective judgment and clinical experience of radiologists, with poor repeatability. However, artificial intelligence radiomics is based on a commercial artificial intelligence software platform, which can quickly screen image data, reduce individual differences and biases, and has strong repeatability.

In this study, a threshold segmentation method (threshold of -350 HU) was employed to consider pulmonary nodules with a solid component ratio of ≥ 50% as mixed-density ground-glass nodules with a predominant solid component. The imaging features are quantified by the full-chest diagnostic module of the Shukun Technology Artificial Intelligence Workstation, and combined with general clinical characteristics to establish a predictive model for the benignancy or malignancy of such pulmonary nodules. This model aims to increase the clinical basis for judging the nature of these nodules and has certain practical value in clinical practice.

## Materials and methods

### Materials

The study was conducted in accordance with the Declaration of Helsinki, and approved by the Institutional Review Board (IRB) of The Fifth Clinical Medical College of Henan University of Chinese Medicine (Zhengzhou People’s Hospital) with a waiver for informed consent (No.2024011155). The study subjects were selected from five medical centers, including Zhengzhou People’s Hospital, Cancer Hospital of the Chinese Academy of Medical Sciences, The First Affiliated Hospital of Zhengzhou University, Liangxiang Hospital in Beijing, and The Ninth People’s Hospital of Zhengzhou. Cases with a confirmed surgical resection and a clear pathological diagnosis of CTR (Cancer-to-Tumor Ratio) greater than 50% were included. These cases were randomly divided into a training set (259 cases) and a validation set (111 cases) in a 7:3 ratio.

### Inclusion criteria

The criteria for the inclusion of subjects in the study are as follows:

1) The maximum diameter of the nodule is ≤ 30 mm;

2) Utilizing the threshold segmentation method applied by Nuance Technology’s artificial intelligence, with a threshold value of -350HU, the 3D CTR is ≥ 50%;

3) Complete preoperative thin-slice chest CT scan data (≤ 1.5 mm) obtained within one month prior to surgery;

4) No evident signs of lung atelectasis, mediastinal lymph node enlargement, or pleural effusion;

5) No prior neoadjuvant therapy, including chemotherapy, targeted therapy, immunotherapy, or radiotherapy;

6) Pathological diagnosis is clearly established postoperatively through routine pathological examination.

### Exclusion criteria

The exclusion criteria for the study are delineated as follows:

1) Nodules with a maximum diameter >30 mm;

2) Cases with a 3D CTR <50%;

3) Non-thin-slice imaging (>1.5 mm) or imaging data obtained more than one month prior to surgery;

4) Subjects who have undergone any form of neoadjuvant therapy;

5) Cases with incomplete medical records or where the pathology is in question.

### Data collection

In total, 370 pulmonary nodules were included in the study, distributed across five medical centers as follows: 182 from Zhengzhou People’s Hospital, 86 from the Cancer Hospital of the Chinese Academy of Medical Sciences, 53 from The First Affiliated Hospital of Zhengzhou University, 38 from Liangxiang Hospital in Beijing, and 11 from The Ninth People’s Hospital of Zhengzhou. Among these, there were 78 benign nodules categorized as follows: 30 inflammatory nodules, 12 hamartomas, 11 tuberculous granulomas, 9 fibrotic nodules, 6 sclerosing hemangiomas, 3 bronchial adenomas, 2 intrapulmonary lymph nodes, 2 cases of carbon deposition, 1 smooth muscle-like hyperplasia, and 1 min meningothelial-like nodule. The malignant nodules numbered 292 and included: 216 invasive adenocarcinomas, 23 minimally invasive adenocarcinomas, 17 mucinous adenocarcinomas, 12 squamous cell carcinomas, 7 carcinomas in situ, 4 small cell lung cancers, 4 metastatic tumors (2 from breast cancer, 1 from thyroid cancer, and 1 from colon cancer), 2 mixed adenocarcinomas, 2 adenosquamous carcinomas, 2 carcinoids, 1 sarcomatoid carcinoma, 1 lymphoepithelioma-like carcinoma, and 1 mucoepidermoid carcinoma.

**Fig. 2:**
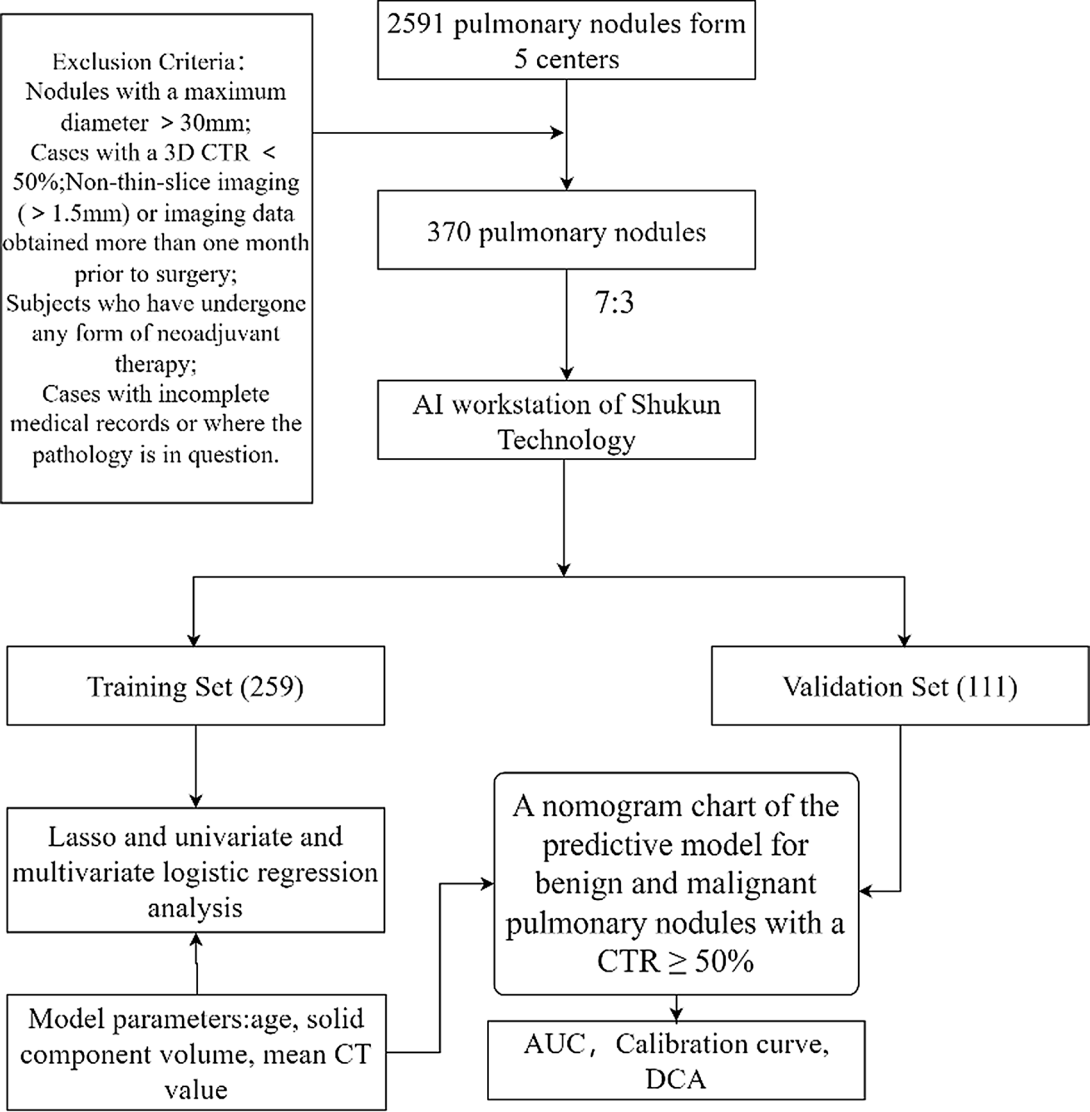
Schematic diagram of the mathematical model of benign and malignant prediction of pulmonary nodules with 3D CTR ≥ 50%

Clinical data encompassed patient age, gender, lobar distribution of the lung, and postoperative pathology. Radiographic characteristics included the volume of solid components and the 3D CTR based on the threshold segmentation method with a threshold of -350HU. Radiomic features, totaling 102, comprised general features, first-order radiomic features, three-dimensional shape features, and texture features (also known as second-order features, which reflect the periodic appearance of gray levels in the image and their spatial relationships, indicating the uniformity, fineness, and roughness of the image, with a total of 5 categories). These included GLCM features, GLSZM features, GLRLM features, NGTDM features, and GLDM features. The detailed content of these features is presented in the third section of this paper. A comprehensive compilation and organization of the aforementioned 108 feature items were conducted. The detailed flowchart is depicted in Fig. 2.

### Statistical methods

The statistical analysis and visualization of the data were conducted using R (version 4.2.1). Initially, a 10-fold cross-validation least absolute shrinkage and selection operator (LASSO) regression method was employed to identify features with non-zero coefficients from both general characteristics and radiomic features. These features were then subjected to univariate logistic regression analysis. Variables with a p-value less than 0.1 in the training set were selected for inclusion in the multivariate logistic regression analysis. Features with a p-value less than 0.05 were incorporated into the benign and malignant prediction model.

The performance of the predictive model was evaluated using the receiver operating characteristic (ROC) curve, decision curve analysis (DCA), and calibration curves. The predictive model was further validated by applying the validation set data to the model. A p-value less than 0.05 was considered to indicate statistical significance.

## Results

### LASSO regression coefficient selection results

**Fig. 3:**
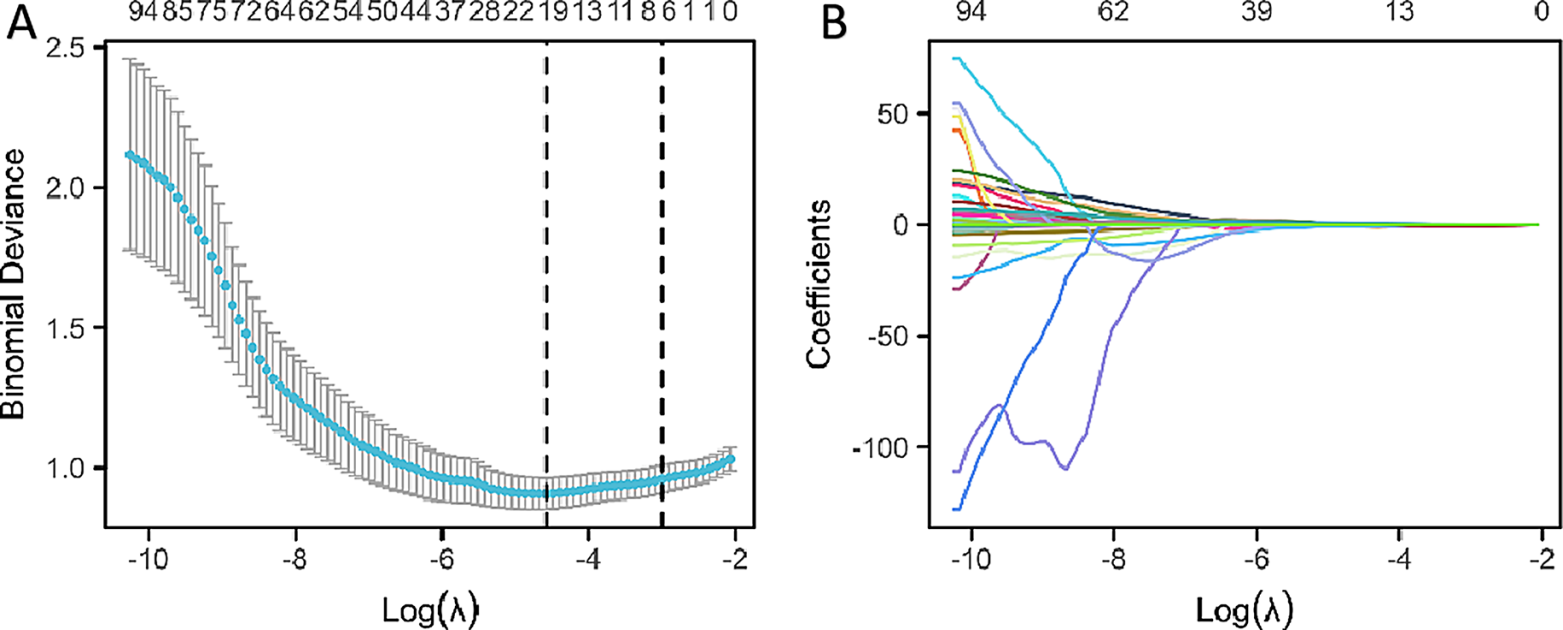
The process of selecting variables in Lasso regression

In the process of feature selection, 18 non-zero coefficient features were identified from the 108 characteristics using the Least Absolute Shrinkage and Selection Operator (LASSO) regression method. These features include: age, lobe, Volume of solid components, Kurtosis, Maximum, Mean, Minimum, sphericity×10, GLCMImc2, GLCM Inverse Variance×10, GLSZM Gray Level Variance, GLSZM Small Area Emphasis×10, GLSZM Small Area Low Gray Level Emphasis×100, GLSZM Zone Entropy, GLRLM Run Variance, NGTGDM Strength, GLDM Large Dependence High Gray Level Emphasis/1000, GLDM Large Dependence Low Gray Level Emphasis. The process of selecting variables in Lasso regression is illustrated in Fig. 3A and B.

### Construction of the clinical factor model

The 18 features identified through LASSO regression were subjected to univariate logistic regression analysis. Factors with a p-value less than 0.1 were selected for inclusion in the multivariate logistic regression analysis. Subsequently, factors with a p-value less than 0.05 were incorporated into the final predictive model. The detailed results of the univariate and multivariate logistic regression analyses are presented in Table 1.

Ultimately, three factors were included in the model: patient age, volume of solid components, and mean CT value. The regression equation for the predictive model is as follows: P = e^x^/(1 + e^x^), x = 2.4182 − 0.0490×(age) − 0.0004×(solid component volume) + 0.0061×(mean CT value).

**Table 1:** Univariate analysis and multivariate analysis

Characteristics	Total(*N*)	Univariate analysis		Multivariate analysis	
		Odds Ratio (95% CI)	*P* value	Odds Ratio (95% CI)	*P* value
age	370	0.953 (0.929–0.977)	**< 0.001**	0.960 (0.934–0.987)	**0.004**
lobe	370	0.870 (0.735–1.031)	0.108		
Volume of solid components	370	1.000 (1.000–1.000)	**0.004**	1.000 (0.999–1.000)	**0.015**
Kurtosis	370	1.055 (0.989–1.125)	0.107		
Maximum	370	1.000 (1.000–1.001)	0.374		
Mean	370	1.004 (1.001–1.006)	**0.003**	1.005 (1.001–1.008)	**0.024**
Minimum	370	1.004 (1.002–1.005)	**< 0.001**	1.001 (0.998–1.004)	0.438
sphericity×10	370	1.607 (1.003–2.574)	**0.049**	0.583 (0.321–1.061)	0.077
GLCM Imc2	370	0.182 (0.017–2.000)	0.164		
GLCM Inverse Variance×10	370	1.349 (0.988–1.843)	0.060	1.873 (0.925–3.790)	0.081
GLSZM Gray Level Variance	370	0.989 (0.980–0.998)	**0.017**	1.005 (0.990–1.020)	0.517
GLSZM Small Area Emphasis×10	370	2.245 (1.406–3.587)	**< 0.001**	2.066 (0.820–5.204)	0.124
GLSZM Small Area Low Gray Level Emphasis×100	370	1.562 (1.227–1.989)	**< 0.001**	1.328 (0.858–2.055)	0.204
GLSZM Zone Entropy	370	0.229 (0.136–0.384)	**< 0.001**	0.520 (0.129–2.101)	0.358
GLRLM Run Variance	370	0.998 (0.534–1.865)	0.996		
NGTGDM Strength	370	1.042 (0.977–1.112)	0.210		
GLDM Large Dependence High Gray Level Emphasis/1000	370	0.993 (0.985–1.002)	0.130		
GLDM Large Dependence Low Gray Level Emphasis	370	1.359 (0.828–2.233)	0.225		

### Training set ROC curve analysis and validation

**Fig. 4:**
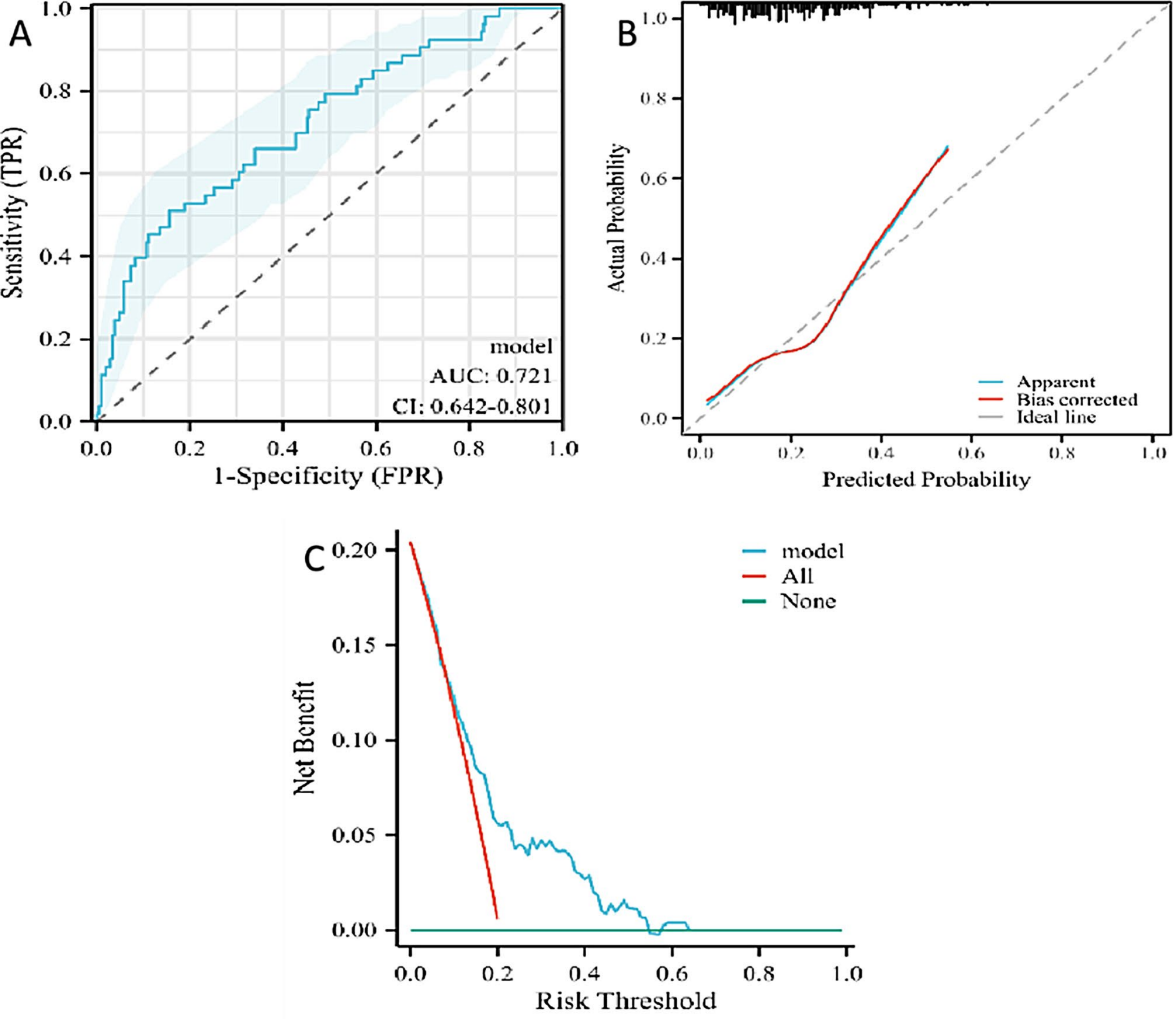
The ability of the model to discriminate benign and malignant sub-solid pulmonary nodules with a 3D CTR ≥ 50% was validated using ROC, calibration, and DCA curves. ROC curves (**A**), calibration curves (**B**), and DCA curves (**C**) of the training cohort

The training set comprised 259 nodules, with 206 benign and 53 malignant cases. The specific data are presented in Table 2. The predictive performance of the model was assessed using the ROC curve, which yielded an AUC of 0.721, indicating moderate accuracy, with a 95%CI ranging from 0.642 to 0.801 (as shown in Fig. 4A). At the cutoff point of -2.7206, the Youden’s index was maximized (0.3541). At this point, the model’s sensitivity was 0.50943, specificity was 0.8447, the positive predictive value was 0.4576, and the negative predictive value was 0.8700.

Calibration curves were used to evaluate the model’s ability to accurately estimate the malignant risk of pulmonary nodules with a CTR greater than 50% within the training set (as shown in Fig. 4B). The analysis indicated that the calibration curve of the training set had a high degree of overlap with the ideal curve, and the discrimination indicated that the model had moderate accuracy (C-index: 0.721 (0.641–0.802)). The calibration curve suggested that there was no significant difference between the predicted and observed values, indicating a good fit of the model (*P* = 0.4669).

Additionally, DCA was employed to evaluate the model (as depicted in Fig. 4C). It indicated that when the threshold probability for intervention ranged from 0.05 to 0.58, the net benefit of the DCA curve was higher than that of the “no intervention” and “full intervention” strategies, suggesting that the model has good clinical utility.

**Table 2:** Details the characteristics of the factors included in the predictive model from the training dataset

Variables	Total (*n* = 259)	Non-invasive group (*n* = 206)	Invasive group (*n* = 53)	Statistic	*P*
Age	59.60 ± 10.00	60.42 ± 9.63	56.42 ± 10.87	t = 2.63	**0.009**
Volume of solid components	1856.05 ± 2083.73	2034.12 ± 2224.55	1163.92 ± 1191.51	t = 3.86	**< 0.001**
Mean CT value	-61.43 ± 121.62	-69.40 ± 124.55	-30.47 ± 104.87	t=-2.31	**0.023**

t: t-test

SD: standard deviation

### Nomogram model construction

**Fig. 5:**
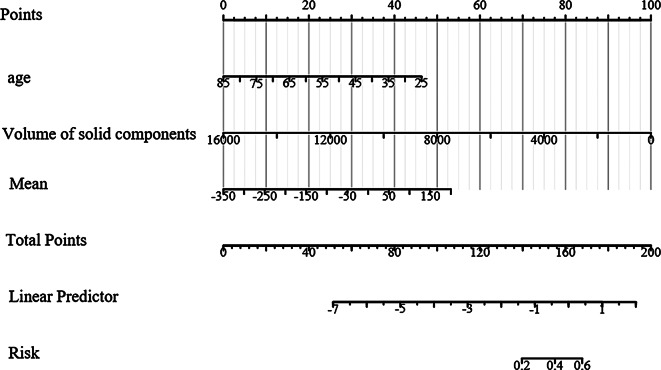
Nomogram model for predicting benign and malignant sub-solid pulmonary nodules with a 3D CTR ≥ 50%

Based on the results of the multivariate logistic regression analysis, which identified three predictive factors incorporated into the model, a nomogram was constructed for visual representation and ease of use (Fig. 5). The nomogram is a graphical tool that integrates the predictive factors and allows for the estimation of the probability of a malignant nodule.

### Validation set ROC curve analysis and validation

**Fig. 6:**
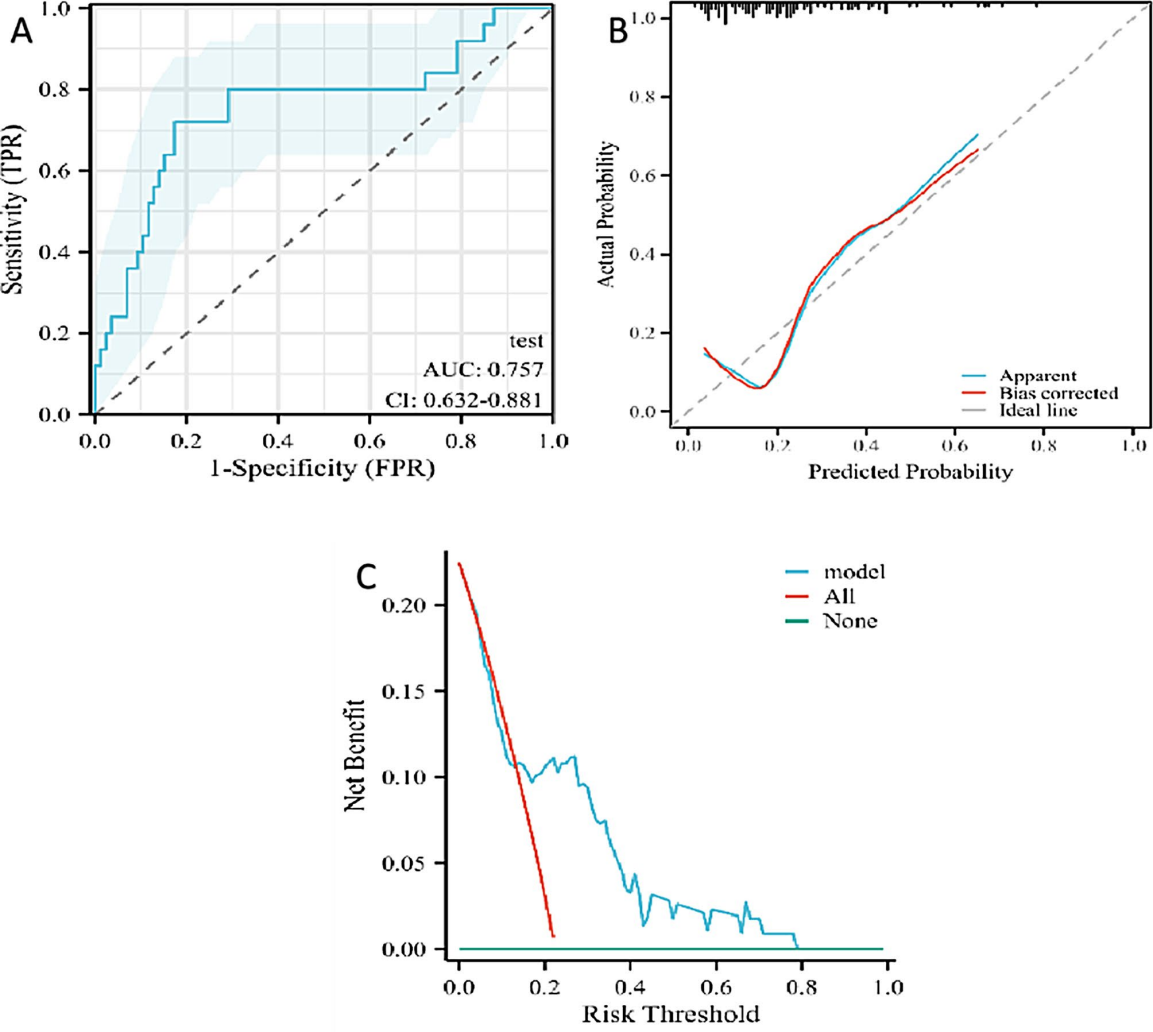
ROC curves (**A**), calibration curves (**B**), and DCA curves (**C**) of the validation cohort

The validation set, consisting of 111 nodules (86 benign and 25 malignant), was used to assess the predictive performance of the model. The comparison between the training and validation sets is detailed in Table 3, where no significant statistical differences were observed for the three factors included in the model (*P* < 0.05). The validation process involved the construction and analysis of the ROC curve, calibration curve, and DCA curve (as depicted in Fig. 6A, B, and C).

The ROC curve for the validation set demonstrated a high predictive performance with an AUC of 0.757 and a 95%CI ranging from 0.632 to 0.881. The sensitivity and specificity of the model in the validation set were 0.8200 and 0.8256, respectively, with an overall accuracy of 0.8018. The calibration curve indicated a C-index of 0.757 (0.632–0.881), suggesting moderate calibration. However, the p-value of 0.0159 indicates a statistically significant difference between the predicted and observed values, suggesting that the model’s calibration may require further refinement.

The DCA curve analysis showed that when the probability threshold for malignancy of pulmonary nodules was between 0.18 and 0.78, the model provided a higher net benefit, indicating its clinical utility within this range of risk probabilities.

**Table 3:** Compares the two groups in the training and validation datasets

Variables	Total (*n* = 370)	test (*n* = 111)	train (*n* = 259)	Statistic	*P*
Age	59.65 ± 10.09	59.75 ± 10.32	59.60 ± 10.00	t = 0.13	0.899
Volume of solid components	1804.36 ± 2011.32	1683.75 ± 1834.60	1856.05 ± 2083.73	t=-0.75	0.451
Mean	-59.00 ± 123.08	-53.32 ± 126.80	-61.43 ± 121.62	t = 0.58	0.562

t: t-test

SD: standard deviation

### Discussion

The early detection and accurate differentiation of pulmonary nodules into benign or malignant categories are crucial for the early diagnosis and treatment of lung cancer. The consolidation tumor ratio (CTR), which is the ratio of solid components to the total nodule volume in pulmonary nodule imaging, is a significant indicator. An increase in solid components within malignant pulmonary nodules typically signifies a higher degree of invasiveness [6, 7]. However, solid pulmonary nodules paradoxically have the lowest probability of malignancy among all types of pulmonary nodules, while mixed-density ground-glass nodules have the highest probability [8–10].

Current clinical research primarily focuses on solid nodules and ground-glass nodules without further stratification based on their solid components. Threshold segmentation is a widely used image segmentation technique in medical imaging that involves selecting one or more gray-scale thresholds to categorize pixels into different classes, typically separating the object of interest from the background. The choice of threshold directly affects the determination of the CTR. When the CTR exceeds 50%, clinical differentiation between benign and malignant nodules becomes particularly challenging. This study aims to establish a nomogram model that combines radiomic features quantified using commercially available artificial intelligence software at our institution with general clinical characteristics. The purpose of this model is to provide additional clinical evidence for the differentiation of benign and malignant pulmonary nodules with a CTR greater than 50%.

During the nodule stage, malignant morphological signs such as spiculated margins and pleural indentation are often not typical, making the differentiation between benign and malignant nodules a clinical challenge that requires attention and improved diagnostic capabilities from researchers and clinicians. Currently, clinical practice primarily relies on traditional imaging examinations, such as CT, to assess the size of the nodule, the presence of spiculated margins, pleural indentation, lobulation, cavitation, air bronchogram, satellite nodules, halo sign, and bronchial cutoff, among other morphological characteristics for preliminary judgment [11–15]. When necessary, contrast-enhanced CT may be used to further assess the enhancement pattern of the nodule [16, 17]. Additionally, blood tumor markers such as Carcinoembryonic Antigen (CEA), Squamous Cell Carcinoma Antigen (SCC), Cytokeratin-19 Fragment Antigen 21 − 1 (CYFRA 21 − 1), and Gastrin-releasing Peptide Precursor (ProGRP) [18], as well as novel liquid biopsy markers like single-cell sequencing [19], seven-antibody detection for lung cancer [20], DNA methylation levels [21], and circulating tumor cells [22], are integrated into clinical diagnostic protocols. PET-CT scans are also utilized for assessment, with a maximum standard uptake value (SUVmax) exceeding 2.5 indicating a potential malignancy. It is important to note that some benign conditions like tuberculosis and inflammatory granulomas can also lead to elevated SUVmax, resulting in false positives [23, 24]. Nevertheless, PET-CT remains a widely used diagnostic tool with relatively high accuracy in non-invasive examinations [25, 26]. Lung MRI has been explored as a radiation-free alternative in recent years, though its clinical application is less common [27, 28], and our experience in this area is limited.

There are studies that focus on the differentiation of solid pulmonary nodules. For instance, Xiaodong Xie [29]conducted a retrospective study involving 132 patients with pathologically confirmed solitary pulmonary nodules (SPNs), analyzing their basic information and spectral CT images. The study demonstrated that spectral CT quantitative parameters and their derived parameters are helpful in the differential diagnosis of benign and malignant solid pulmonary nodules. Similarly, Xiao-Qun He [30] retrospectively analyzed CT data from 794 patients with small solid solitary pulmonary nodules (SSPNs) ≤ 15 millimeters in diameter. The nodules were categorized into benign and malignant groups, with each group further divided into three cohorts based on size: Cohort I (diameter ≤ 6 millimeters), Cohort II (6 millimeters < diameter ≤ 8 millimeters), and Cohort III (8 millimeters < diameter ≤ 15 millimeters). Significant differences were observed in the inter-group comparison of polygonal shape and upper lobe distribution in Cohort I, while in Cohort II, polygonal shape, lobulation, pleural indentation, and air bronchogram showed significant differences. In Cohort III, 12 CT features (polygonal shape, calcification, halo sign, satellite nodules, lobulation, air cavity, pleural indentation, bronchial cutoff, and air bronchogram) exhibited significant inter-group differences. Gao Liang [31] developed a radiomics model based on monochromatic dual-energy CT(DECT) images to identify solitary pulmonary nodules with a AUC of 0.8772 (95% CI 0.780–0.974). These findings highlight that CT features may vary among solid pulmonary nodules of different sizes, and recognizing size-specific CT characteristics can aid in minimizing ambiguity and distinguishing benign solid pulmonary nodules from malignant ones. More nuanced differentiation of solid pulmonary nodules has clinical value. However, as of now, pathological diagnosis obtained through CT-guided biopsy or bronchoscopy with navigational bronchoscopic biopsy remains the gold standard for diagnosis.

Lung cancer risk prediction models have seen some clinical application in recent years, primarily based on traditional diagnostic factors such as CT morphological features, tumor markers, smoking history, and family history of cancer. However, the reproducibility of these models is poor, and the varying diagnostic skills of different clinicians can affect the accuracy of clinical applications. Imaging omics features are highly valued for their stability; however, traditional extraction relies on manual delineation of regions of interest, a process that is time-consuming and susceptible to subjective bias. With the application of artificial intelligence in the field of imaging omics, the extraction of some common imaging omics features has become more automated and efficient, thereby enhancing the convenience and accuracy for clinical applications. AI has been increasingly applied in the field of pulmonary nodule diagnosis and treatment in recent years, reducing the workload of radiologists and decreasing misdiagnosis rates. It has also been explored for the detection, diagnosis, and prognosis prediction of solid pulmonary nodules [32–35].

This article introduces a study on solid pulmonary nodules with a solid component ratio greater than 50%, determined by an AI-based threshold segmentation method (threshold − 350HU). The study involved 108 general clinical features and basic radiomic features, which were analyzed using Lasso regression and univariate and multivariate logistic regression. The findings suggest that the volume of solid components and the mean CT value, in combination with patient age, have significant diagnostic value for such nodules. The AUC was 0.721, indicating moderate accuracy, with a 95%CI of 0.642–0.801. The validation set showed an AUC of 0.757 (95% CI: 0.632–0.881). Additionally, a nomogram was constructed based on the three predictive factors, which may have clinical utility.

However, this study has certain limitations. For instance, it did not include detailed clinical risk factors, PET-CT, and morphological features, which could further enhance the precision of the model. Although this study utilized data from multiple centers, the heterogeneity in image acquisition across different centers may limit the model’s generalizability. Furthermore, the dataset is confined to a specific patient population, which may affect the model’s applicability to a broader range of individuals. Future research should consider incorporating a more diverse patient population and varying image acquisition conditions to enhance the model’s ability to generalize. These limitations should be addressed in future research.

### Conclusion

In summary, the diagnosis of predominantly solid pulmonary nodules (CTR>50%) is a clinical challenge. As the detection rate of pulmonary nodules increases, these nodules require more attention from clinicians. Clinical diagnosis based solely on morphological features is often difficult. We have established the nomogram model by defining subsolid nodules using a novel approach based on threshold segmentation with a 3D CTR of at least 50%. The clinical prediction model established in this study may have some clinical application value.

## Data Availability

The datasets used during the current study available from the corresponding author on reasonable request.
